# No evidence of major effects in several Toll-like receptor gene polymorphisms in rheumatoid arthritis

**DOI:** 10.1186/ar2589

**Published:** 2009-01-13

**Authors:** Olivier Jaen, Elisabeth Petit-Teixeira, Holger Kirsten, Peter Ahnert, Luca Semerano, Céline Pierlot, Francois Cornelis, Marie-Christophe Boissier, Geraldine Falgarone, the European Consortium on Rheumatoid Arthritis Families

**Affiliations:** 1 EA-4222, University of Paris 13, 74 rue Marcel Cachin, 93017 Bobigny cedex, Paris, France; 2 Genhotel EA-3886, University Evry-Paris 7 Medical School, Member of the AutoCure European Consortium, CP5727, 91057 Evry-Genopole cedex, Paris, France; 3 University of Leipzig, D-04109 Leipzig, Germany; 4 Rheumatology Department, Avicenne Hospital AP-HP, 93009 Bobigny cedex, Paris, France; 5 Unité de Génétique Clinique, Pôle des Laboratoires Médicaux-Imagerie-Pharmacie, Lariboisière Hospital, AP-HP, 2 rue Ambroise Paré, 75010 Paris, France

## Abstract

**Introduction:**

The objective was to study the potential genetic contribution of Toll-like receptor (TLR) genes in rheumatoid arthritis (RA). TLRs bind to pathogen-associated molecular patterns, and *TLR *genes influence both proinflammatory cytokine production and autoimmune responses. Host–pathogen interactions are involved in RA physiopathology.

**Methods:**

We tested SNPs of five *TLR *genes (*TLR9*, *TLR2*, *TLR6*, *TLR1*, and *TLR4*) in a cohort of 100 French families with RA. Genotypes were analyzed using the transmission disequilibrium test. As *TLR2*, *TLR6*, and *TLR1 *are located on chromosome 4, we determined the haplotype relative risk. Analyses were performed in subgroups defined by status for rheumatoid factor, anti-cyclic citrullinated peptide autoantibodies, and erosions.

**Results:**

We found no disequilibrium in allele transmission for any of the SNPs of the five *TLR *genes. In subgroup analyses, no associations were detected linking *TLR9*, *TLR2*, or *TLR9*/*TLR2 *to rheumatoid factor, anti-cyclic citrullinated peptide autoantibodies, or erosions. Haplotype analysis of the polymorphisms showed no haplotype associations in any of the subgroups.

**Conclusions:**

We found no evidence of major effects of TLR gene polymorphisms in RA, although we tested different *TLR *phenotypes. Moreover, no associations were noted with autoantibody production or erosions.

## Introduction

Rheumatoid arthritis (RA), the most common inflammatory joint disease, exacts a huge toll of disability, deformities, quality-of-life alterations, premature deaths, and economic costs [1]. RA is an autoimmune disease characterized by chronic inflammation of the synovial membrane, which is infiltrated by activated immune cells including CD4^+ ^T cells, B cells, and antigen-presenting cells such as dendritic cells and macrophages. The factors responsible for RA induction and progression are poorly understood but may involve interactions between innate and adaptive immunity [2]. It has been suggested that viruses and bacteria may contribute to initiate or exacerbate RA by binding to Toll-like receptors (TLRs). TLRs are expressed by a variety of immune cells, including B lymphocytes and T lymphocytes, antigen-presenting cells, regulatory T cells and nonimmune cells such as fibroblastic synoviocytes [3-7]. All of these cell populations are found in the rheumatoid synovium. TLR ligands such as peptidoglycans and double-stranded DNA are also present in the rheumatoid synovium [8], suggesting that innate immunity may be involved in initiating the inflammatory process or in inhibiting regulation mechanisms that normally prevent chronic inflammation.

*TLR *gene polymorphisms have been tested in several cohorts. A study of Asp299Gly (rs4986790) and Thr399Ile (rs4986791) *TLR4 *polymorphisms in a cohort of RA patients in Spain found no associations with susceptibility to RA [9]. A case–control study of *TLR4 *Asp299Gly in a cohort in Northern England also found no association, even in the subgroup of patients negative for the shared epitope [10]. Interestingly, heterozygous Asp299Gly status was protective in early untreated RA in a case–control study performed in The Netherlands [11]. Finally, the Asp299Asp polymorphism was associated with higher remission rates after treatment with disease-modifying antirheumatic drugs, compared with the Asp299Gly polymorphism [12]. The role for *TLR4 *in RA, despite studies in various cohorts, therefore remains unclear.

The *TLR2 *polymorphisms Arg677Trp (no rs number reported) and Arg753Gln (rs5743708), both implicated in susceptibility to infection, were not associated with arthritis in a cohort in Spain [9]. Of note, these two *TLR2 *gene mutations were associated with reduced induction of IL-10 and IL-12 expression after stimulation [13]. In mice injected with TLR2 ligands, regulatory T cells lose their regulatory capacities, suggesting a role for *TLR2 *in regulatory T cell control [14]. Prolonged regulatory T cell stimulation by TRL2 ligands may therefore trigger or exacerbate autoimmune responses. Studies in animal models have established that the *TLR2 *status influences the outcome of adjuvant-induced arthritis and streptococcal cell wall arthritis. Mice deficient in MyD88, the TLR adaptor molecule, do not develop arthritis. Similarly, TLR2-deficient mice exhibit lower arthritis scores [15].

As TLR1 and TLR6 are TLR2 co-receptors that increase the number of ligands and induce different transduction pathways [16-19], it was of interest to determine whether the *TLR1 *and *TLR6 *genes showed polymorphisms that were linked to RA. These polymorphisms have been studied in inflammatory bowel disease [20] but not in joint disease. Studies have shown that *TLR1 *Arg80Thr (rs5743610), Asn248Ser (rs4833095), and Ser602Ile (rs5743618) SNPs are associated with invasive aspergillosis [21] and with Crohn's disease [20]. The *TLR6 *Ser249Pro SNP (rs5743810) is associated with a reduced risk of asthma and an increased risk of invasive aspergillosis [21,22].

Transcription factors that bind to the *TLR9 *promoter region include GATA-1, GATA-2, c-Ets, and CP2 [23]. *In silico *investigations indicate that the -1486 T/C (rs187084) substitution probably creates an SP-1 binding site [24]. Nevertheless, neither this SNP nor *TLR9 *+2848A/G (rs352140) was associated with systemic lupus erythematosus in a cohort in Korea [23]. In contrast, the TLR9 -1237C/T (rs5743836) polymorphism was associated with Crohn's disease [25] and with asthma [26].

In the present article, we investigated potential associations between RA and SNPs of *TLR1*, *TLR2*, *TLR4*, *TLR6*, and *TLR9 *in a cohort of French Caucasian families with RA. We elected to investigate a range of TLRs believed to interact with viruses, Gram-positive bacteria, or Gram-negative bacteria. We used PCR-RFLP and matrix-assisted laser desorption/ionization–time of flight mass spectrometry to determine the genotypes of 100 family trios, each comprising the index patient and both healthy parents. We analyzed several subgroups of severe RA that might be linked to *TLR *gene polymorphisms, including the subgroups with rheumatoid factor (RF) or with anti-cyclic citrullinated peptide antibody (anti-CCP), two RA-related autoantibodies, and the subgroup with joint erosions, since these are often associated with autoantibody production.

## Materials and methods

### Demographic and clinical features of the study population

RA families were recruited through a national media campaign followed by selection of individuals who fulfilled the 1987 American College of Rheumatology revised criteria for RA. A rheumatologist reviewed all clinical data. In each of 100 French Caucasian families, we studied one individual with RA and both parents; to be eligible for the study, all four grandparents of the patient had to be European Caucasians. Among the 100 RA patients, 87 were women and 13 were men; their mean age at disease onset was 39.6 years, 72% were RF-positive, 81% were anti-CCP-positive, 86% exhibited joint erosions, and 90% had rheumatoid nodules. All study participants provided informed consent, and the appropriate ethics committee (Bicêtre Teaching Hospital, Paris, France) approved the study.

### Molecular genotyping methods

Genomic DNA was purified from fresh peripheral blood leukocytes using standard methods. To genotype *TLR *genes, we performed PCR-RFLP analysis or single-base extension followed by mass spectrometry.

For PCR-RFLP analysis, each amplification on each sample was performed in a 35 μl reaction volume composed of 10× PCR buffer (Perkin Elmer, Boston, MA, USA), 0.5 μM each primer, 0.1 mM each dNTP, 1.25 units Taq Gold DNA polymerase (Perkin Elmer), 3 mM MgCl_2_, and 70 ng genomic DNA, diluted to the final volume with H_2_O.

#### Toll-like receptor 1

Three SNPs were genotyped for TLR1. SNP1 (rs5743618) is a G/T polymorphism of Ser602Ile in exon 4 at position +7765 of the gene. SNP2 (rs5743594) is a C/T polymorphism constituting a noncoding mutation in intron 2 at position +3663. SNP3 (rs5743560) is an A/C polymorphism constituting a noncoding mutation in intron 1 at position +214; this SNP did not exhibit sufficient polymorphism in the participants to allow genotyping in the association study.

The primers used for PCR amplification are presented in Table 1. Conditions will be supplied on request.

**Table 1:** Comparison of SNPs in the present study with the literature (Reference SNP (rs) and PCR primers)

PubMed identifier	Gene	SNP reference	Allele	MAF^a^	Found in the literature?	PCR primers
Present study	*TLR1*	rs5743618	G/T		No	Forward, 5'-CCCGGAAAGTTATAGAGGAACCCT-3'
						Reverse, 5'-TTCACCCAGAAAGAATCGTGCCCA-3'
Present study		rs5743594	C/T		No	Forward, 5'-AAGATCAGGGTGGTAGTGTTGG-3'
						Reverse, 5'-CCCAATTCTTCCTCTCCAGCTT-3'
Present study	*TLR2*	rs3804099	C/T		No	Forward, 5'-ATCGTCTTCCTGGTTCAAGC-3'
						Reverse, 5'-CAGTTCCAAACATTCCACGG-3'
Present study		rs4696480	T/A		No	Forward, 5'-CAAATTTAAAAGAGGGCAAGAAA-3'
						Reverse, 5'-CAGTTTATTGTGAGAATGAGTTT-3'
Present study	*TLR4*	rs2737191	A/G		No	Forward, 5'-CATCCCCTACTTTCTTCACA-3'
						Reverse, 5'-TCAACTCAGGACCCATAATC-3'
Present study		rs4986790	A/G	32%/32.50%	Yes: other name Asp299Gly	Forward, 5'-TCTGGGAGAATTTAGAAATGAA-3'
						Reverse, 5'-AAACGTATCCAATGAAAAGAAG-3'
Present study		rs1554973	T/C		No	Forward, 5'-CAAAGGATATGTGAACAATAGG-3'
						Reverse, 5'-AATCCCGTGAGTAGAGAATG-3'
Present study	*TLR6*	rs5743810	C/T		No	Forward, 5'-ACTTGGTTCGTGATATGTTCTA-3'
						Reverse, 5'-AAACCCTTCACCTTGTTTTTCA-3'
Present study	*TLR9*	rs187084	C/T		No	Forward, 5'-TCTGGGACAAGTCCAGCCAG-3'
						Reverse, 5'-GGACACTCCCAGCTCTGAAG-3'
Present study		rs352140	T/C		No	Forward, 5'-CTGCTAGCACACCGGATCAT-3'
						Reverse, 5'-ATGATACCACCCAGAGTGGG-3'

^a^Mutation allele frequency, presented as cases/controls.

#### Toll-like receptor 2

Five SNPs were initially chosen for TLR2. These SNPs were rs1816702 (SNP1), rs3804099 (SNP2), rs5743708 (SNP3), rs1804965 (SNP4), and rs4696480 (SNP5). SNP1 is a C/T polymorphism that constitutes a noncoding mutation in intron 1 at position +458 of the gene. Primers used for the SNP1 PCR arepresented in Table 1. Conditions will be supplied on request for all SNPs. SNP2 is a C/T polymorphism that constitutes a synonymous coding mutation in exon 2 at position +15591 of the gene. SNP3, SNP4, and SNP5 of *TLR2 *did not exhibit sufficient polymorphism for evaluation using the transmission disequilibrium test (TDT).

#### Toll-like receptor 4

Three SNPs were selected for genotyping TLR4 based on location within the gene, validation status, and minor allele frequency. SNP1 (rs4986790) is an A/G SNP where A is the ancestral allele with a frequency of about 95% in Caucasian populations. It is located in exon 4 and leads to the amino acid change D299G. SNP2 (rs2737191) is an A/G SNP where A is the ancestral allele with a frequency of 70% to 80% in Caucasians. This SNP is located upstream of *TLR4*. SNP3 (rs1554973) is a C/T SNP. The ancestral allele C has a frequency of 20% to 30% in Caucasians.

Genotyping was carried out essentially as described previously (PubMed Identifier: 17160404). Assay design was supported by Calcdalton software (PubMed Identifier: 16526404). For each SNP, both PCR-RFLP and single-base extension were performed. The primers are presented in Table 1. PCR conditions will be supplied on request.

#### Toll-like receptor 6

The TLR6 SNP1 and SNP2 were rs5743810 and rs5743795, respectively. SNP1 is a C/T polymorphism that constitutes a Ser249Pro coding mutation in exon 1 at position +744 of the gene. SNP2 is a G/A polymorphism that is a noncoding mutation located in the presumptive promoter region, at position -1335.

The primers used for PCR amplification of SNP1 and SNP2 are presented in Table 1. PCR conditions will be supplied on request. For SNP2, the family genotypes contained only G alleles, indicating that this SNP did not exhibit sufficient polymorphism for evaluation using the TDT set 1.

#### Toll-like receptor 9

TLR9 SNP1 was rs187084 and TLR9 SNP2 was rs352140. SNP1 is a C/T polymorphism that is a synonymous P545P coding mutation on exon 2 at position +2848 (reference genomics) but at position +3483 of the gene (+1 being at the beginning of exon 1 instead of exon 2). SNP2 is a C/T polymorphism that constitutes a noncoding mutation, probably at position -1486 of the promoting region but at position -851 of the gene (+1 being at the beginning of exon 1 instead of exon 2).

The primers used for PCR amplification of SNP1 and SNP2 PCR are presented in Table 1. PCR conditions will be supplied on request.

### Linkage and association analysis

The Hardy–Weinberg equilibrium was checked in control individuals, using a chi-square test with one degree of freedom. The linkage analysis relied on the TDT, in which the observed transmission of a specific allele from heterozygous parents to RA patients is compared with the transmission predicted based on Mendelian inheritance (50%) [27]. For the association analysis, we used the genotype relative risk, which compares the genotype in the affected offspring with the control genotype derived from untransmitted parental chromosomes. *P *< 0.05 was considered statistically significant.

### Power calculation

The power calculation was estimated as described elsewhere [28] and was calculated as follows. Based on twin studies, genetic factors of RA are estimated as 30% [29] to 60% [30,31]; since 50% is the value commonly accepted, genetic variance was then estimated to be 0.5. Environmental factors are estimated to explain 40% to 50% of the disease [32,33]; the genetic variance that has therefore been maintained is 0.4. For the locus variance, no formal data are available. We decided to keep the less favorable value for a minor participation of the gene; this encouraged us to keep a variance for the locus of 0.05 to 0.2. The power finally estimated was 80% for a number of families needed of 67 with the "TDT power calculator".

## Results

### Hardy–Weinberg equilibrium check

In the control samples composed of the parental alleles that were not transmitted to RA patients, all tested SNPs were in Hardy–Weinberg equilibrium.

### Association studies in the overall population

None of the alleles of any of the 10 SNPs showed disequilibrium of transmission to RA patients by the TDT (Table 2). Neither did the genotype relative risk indicate any genotype associations with RA (Table 3). The study allowed detection of risk factors with allelic odds ratios ranging from 1.76 to 2.85 (corresponding to frequency differences between cases and controls of 14% to 8%) and of protective factors with allelic odds ratios ranging from 0.08 to 0.56 (corresponding to frequency differences of 4.6% to 13.8%; Table 4).

**Table 2:** Results of the transmission disequilibrium test in 100 French Caucasian families with rheumatoid arthritis

Gene	SNP reference (position and amino acid change)	Allele	Transmitted/untransmitted	*P *value
*TLR1*	rs5743618 (+7765 S602I)	G/T	41/35	0.49
	rs5743594 (+3663)	C/T	21/24	0.65
*TLR2*	rs3804099 (+15591 N199N)	C/T	52/46	0.54
	rs4696480 (-1938)	T/A	49/52	0.76
*TLR4*	rs2737191 (-3869)	A/G	45/44	0;92
	rs4986790 (+8719 D259G)	A/G	10/12	0.67
	rs1554973 (+14229)	T/C	33/34	0.9
*TLR6*	rs5743810 (+744 S249P)	C/T	58/54	0.71
*TLR9*	rs187084 (+3483 P545P)	C/T	43/42	0.91
	rs352140 (851)	T/C	40/42	0.83

**Table 3:** Results of the genotype relative risk test in 100 French Caucasian families with rheumatoid arthritis

Gene	SNP reference	Genotype	Patients (n = 100)	Controls (n = 100)	*P *value
*TLR1*	rs5743618 (+7765 S602I)	GG	52	49	0.67
		GT	44	44	
		TT	4	7	
	rs5743594 (+3663)	CC	71	71	0.41
		CT	25	28	
		TT	4	1	
*TLR2*	rs3804099 (+15591 N199N)	CC	29	24	0.77
		CT	51	55	
		TT	20	21	
	rs4696480 (-1938)	TT	18	21	0.89
		TA	56	53	
		AA	26	26	
*TLR4*	rs2737191 (-3869)	AA	46	47	0.29
		AG	44	41	
		GG	10	12	
	rs4986790 (+8719 D259G)	AA	89	90	1
		AG	10	10	
		GG	1	0	
	rs1554973 (+14229)	TT	56	58	0.88
		TC	39	36	
		CC	4	5	
*TLR6*	rs5743810 (+744 S249P)	CC	22	20	0.91
		CT	55	55	
		TT	23	25	
*TLR9*	rs187084 (+ 3483 P545P)	CC	18	16	0.89
		CT	45	48	
		TT	36	35	
	rs352140 (-851)	TT	34	24	0.36
		TC	37	47	
		CC	28	28	

**Table 4:** Effect size of allelic associations detectable in our study (100 cases and 100 matched controls)

Gene	SNP reference	Risk effect	Protective effect
*TLR1*	rs5743618	>1.80 (+13.4%)	<0.51 (-11.8%)
	rs5743594	>2.03 (+11.4%)	<0.38 (-8.6%)
*TLR2*	rs3804099	>1.76 (+13.9%)	<0.56 (-13.8%)
	rs4696480	>1.76 (+13.9%)	<0.56 (-13.8%)
*TLR4*	rs2737191	>1.78 (+13.7%)	<0.53 (-12.3%)
	rs4986790	>2.85 (+8%)	<0.08 (-4.6%)
	rs1554973	>1.86 (+12.8%)	<0.53 (-9.3%)
*TLR6*	rs5743810	>1.76 (+13.9%)	<0.56 (-13.7%)
*TLR9*	rs187084	>1.76 (+14%)	<0.55 (-13.2%)
	rs352140	>1.76 (+13.9%)	<0.56 (-13.8%)

Effect sizes presented as allelic odds ratios (frequency differences between cases and controls) for the minor alleles.

### Association studies in the subgroups with rheumatoid factor, anti-cyclic citrullinated peptide antibodies, or erosions

As TLRs have adjuvant effects on B cells and T cells to promote the antibody response, we separately evaluated patient subgroups defined by the presence of anti-CCP antibody and/or RF. In neither subgroup did we find any associations between RA and *TLR1*, *TLR2*, *TLR4*, *TLR6*, or *TLR9 *SNP alleles (Table 5). Finally, none of the alleles was associated with RA in the subgroup of patients who had joint erosions (Table 5).

**Table 5:** Transmission disequilibrium test in rheumatoid arthritis families with RF, anti-CCP antibody, or joint erosions

		RF		Anti-CCP antibody		Erosions	
				
Gene	SNP reference	Transmitted/untransmitted	*P *value	Transmitted/untransmitted	*P *value	Transmitted/untransmitted	*P *value
*TLR1*	rs5743618 (+7765 S602I)	33/28	0.52	34/29	0.53	32/32	1
	rs5743594 (+3663)	15/22	0.25	14/20	0.3	20/21	0.88
*TLR2*	rs3804099 (+15591 N199N)	39/40	0.91	41/36	0.57	47/39	0.39
	rs4696480 (-1938)	46/39	0.45	41/44	0.74	42/48	0.53
*TLR4*	rs2737191 (-3869)	38/35	0.73	33/36	0.72	38/42	0.65
	rs4986790 (+8719 D259G)	7/9	0.62	9/12	0.51	10/11	0.83
	rs1554973 (+14229)	30/30	1	25/25	1	32/31	0.9
*TLR6*	rs5743810 (+744 S249P)	48/41	0.46	48/44	0.68	51/49	0.84
*TLR9*	rs187084 (+3483 P545P)	37/35	0.81	38/35	0.73	40/39	0.91
	rs352140 (-851)	36/37	0.91	34/36	0.81	37/35	0.81

RF, rheumatoid factor; anti-CCP, anti-cyclic citrullinated peptide.

### *TLR1 *and *TLR6 *haplotype study

As *TLR1 *and *TLR6 *genes are located in the same region of chromosome 4, we performed TDTs on haplotypes whose frequency was greater than 5%. Although these receptors function with the same co-receptor TLR2, we found no association between the haplotype and RA (Table 6), even when we confined our analysis to the subgroups with RF or with anti-CCP antibody.

**Table 6:** Transmission disequilibrium test for *TLR1–TLR6 *haplotypes (rs5743618–rs5743594–rs5743810)

	Overall population		RF-positive patients		Anti-CCP-positive patients	
			
Haplotype (frequency >5%)	Transmitted/untransmitted	*P *value	Transmitted/untransmitted	*P *value	Transmitted/untransmitted	*P *value
GCC	16/12	0.45	13/8	0.28	12/9	0.51
GCT	28/31	0.7	21/27	0.39	22/26	0.56
GTC	12/11	0.83	10/7	0.47	9/7	0.62
TCC	20/21	0.88	18/20	0.75	18/19	0.87

Linkage disequilibrium: TLR1_SNP1 (rs5743618) – TLR1_SNP2 (rs5743594), *D*' = 0.94; TLR1_SNP2 (rs5743594) – TLR6_SNP1 (rs5743810), *D*' = 0.5. RF, rheumatoid factor; anti-CCP, anti-cyclic citrullinated peptide.

### General situation for TLR-gene association with RA and comparison for TLR4

A PubMed search for TLRs and RA yields was performed, and we found nine papers on the subject. In these nine articles, populations are small and diverse; it appears that there is conflicting evidence for association of TLR4 polymorphisms but not conclusive evidence for any association of previously described polymorphisms with RA. These studies are listed in Tables 1 and 7. This analysis reinforces the community relevance of our data on TLR SNPs.

**Table 7:** Overview of Asp299Gly in TLR4: interesting deviations in allele frequencies

PubMed identifier	Gene	SNP reference	Allele	MA	MAF^a^	Odds ratio (*P *value)	Population	Articular disease
Present study	*TLR4*	rs4986790	A/G	G	6%/5.0%		100 trios, French	RA
17143969	*TLR4*	rs4986790	A/G	G	7.5%/2.6%	3.1 (0.037)	CC 101/100, Canadian	AS
16837493	*TLR4*	rs4986790	A/G	G	15%/14.4%		CC 193/125, Scottish	AS
16567359	*TLR4*	rs4986790	A/G	G			CC AS138, ReA91/140	AS
15647432	*TLR4*	rs4986790	A/G	G		1.68	CC 113/170, Dutch	AS
15498795	*TLR4*	rs4986790	A/G	G	5%		313 trios, UK	JA
15022344	*TLR4*	rs4986790	A/G	G	5.3%/8.6%		CC 282/314, Dutch	RA
NCBI-SNP	*TLR4*	rs4986790	A/G	G	?/3.30%		CEU	N/A
NCBI-SNP	*TLR4*	rs4986790	A/G	G	?/0		HCB	N/A
NCBI-SNP	*TLR4*	rs4986790	A/G	G	?/0		JPT	N/A
NCBI-SNP	*TLR4*	rs4986790	A/G	G	?/3.30%		YRI	N/A

^a^Presented as cases/controls. NCBI, National Center for Biotechnology Information; CEU, Utah residents with ancestry from northern and western Europe; HCB, Han Chinese in Beijing; JPT, Japanese in Tokyo; YRI, Yoruba in Ibadan, Nigeria; MA, mutation allele; MAF: mutation allele frequency; CC, case control study; RA, rheumatoid arthritis; AS, ankylosing spondylitis; JA, juvenile arthritis; N/A, not applicable.

## Discussion

We did not observe a large effect of the *TLR1*, *TLR2*, *TLR4*, *TLR6*, or *TLR9 *genes in a cohort of French Caucasian families with RA. The present study was properly designed since we chose the candidate genes before performing the linkage/association analysis. We used the TDT, which simultaneously evaluates linkage and association, thus avoiding biases due to the inevitable imperfections in matching between cases and controls. Moreover, we had a high homogeneous cohort where all the patients had four European Caucasian grandparents.

To our knowledge, this is the first study of *TLR1*, *TLR6*, and *TLR9 *in a cohort with RA. TLR1 and TLR6 are co-receptors but might display specific polymorphisms, no evidence of which was found in our cohort. TLR9 is involved in autoantibody production, as shown in the model developed by Leadbetter and colleagues [34], and probably but indirectly in inactivated DNAse mice [35]. Whether the role for TLR is confined to autoantibody production remains unclear; TLR may exert key effects on interactions between B cells and T cells, as well as on T-cell regulation mechanisms. For this reason, we performed subgroup analysis in patients with RF, anti-CCP antibodies, or joint erosions – and found no effect. Even if this stratification reduces the number of investigated patients, each feature investigated is extremely frequent and so the subgroups maintain a numerosity comparable with the main sample. Moreover, subgroup analysis is justified by the fact that RA is a complex disease that reasonably might have different etiopathogenesis subgroups. If a subgroup matches a certain etiopathogenesis, then the effect size of a genetic variant might be much higher than for the whole RA population on average.

The polymorphisms tested in our study were selected based on frequency and on feasibility of tests; neither their location in exons or introns nor the nature of the polymorphisms was a selection criterion. Sequences located in intron or promoter regions hold appeal for research, because chromosomal interactions occur between genes independently from enhancer sequences known to exist in the regulated gene. Independent genes can therefore exert effects via intra-chromosomal and inter-chromosomal interactions during cell activation [36]. As TLR1 and TLR6 act as co-receptors with TLR2 and are promiscuous in the genome, we performed haplotype analysis for the *TLR1 *and *TLR6 *genes. We found no associations between the frequent haplotypes and RA susceptibility in the overall group or in subgroups defined by the presence of RF, anti-CCP antibodies, or joint erosions (data not shown).

Because RA patients were eligible for our study only if both their parents were alive, our RA population contains an unusually high proportion of young patients. Conceivably, this bias toward younger patients may have led to unusually high prevalences of criteria for severe RA (RF, anti-CCP antibodies, and rheumatoid nodules), since these allow a definitive diagnosis early in the course of the disease. Our working hypothesis that *TLR *genes might be associated with greater disease severity by increasing autoantibody production received no support from our findings.

Definitive proof that autoantibodies are involved in the pathophysiology of RA is still lacking, despite accumulating evidence of a role for B cells – including the efficacy of second-line treatments targeting B cells in severe RA [37]. B cells express *TLR *and may exert pathogenic effects in RA after TLR stimulation, independently from autoantibody production, since they are involved in presenting autoantigens to T cells, producing cytokines, and inducing ectopic architecture [38]. Furthermore, there is strong evidence of a lack of tolerance in RA, which may be ascribable to regulatory-T-cell impairment at the time of TLR2 stimulation with TLR1 or TLR6 co-engagement by ligands, allowing pathogenic immune cells to escape from normal regulatory mechanisms and to trigger or exacerbate arthritis. Moreover, TLR9 engagement was shown to induce a T-helper type 1 isotypic switch in B lymphocytes, which may be involved in the pathogenesis of RA. Further evidence of the role for TLR9 comes from the efficacy in RA of chloroquine and quinacrine, both of which block TLR9 signaling in antigen-presenting cells [39]. Finally, studies on human rheumatoid tissue indicate that TLRs play a potential role in driving inflammation and/or destructive process in RA [40]. In our cohort, we found no evidence that the *TLR *polymorphism influenced the above-described effects.

We found no associations between RA and *TLR *polymorphisms in more severe subgroups – with RF, or anti-CCP antibodies, or joint erosions – in our cohort of French Caucasians. Similarly, studies conducted in Spain [9] and in England [10] showed no associations between RA and *TLR4 *or *TLR2*. A statistically significant decrease in the G allele of *TLR4 *Asp299Gly (rs4986790) was noted in RA patients in a case–control study in The Netherlands [11]. In contrast, in our study there was no significant G-allele enrichment in the RA patients. Our study had a power of 74% to detect a difference at least as great as the one found in the study from The Netherlands [11]. Our results therefore rule out a protective role for *TLR4 *Asp299Gly in our French Caucasian cohort. Nevertheless, although *TLR4 *may not be involved in initiating RA, it seems to be important in the early treatment response. Remission rates are therefore higher in patients with the A896A genotype than in patients with the uncommon G896G genotype or in heterozygous patients [12].

In conclusion, the role for *TLR4 *in the pathogenesis of RA remains uncertain. Altogether, published data and our data lead to the prediction that, to improve these data, analyses in larger cohorts with more than 500 patients would be required. Since this sample size is not easy to reach in single-center or even multicenter studies, meta-analytic analysis could probably be the only feasible approach. With that method, our results might help to spread light on the overall contribution of *TLR *genes in RA.

We tested five out of the 11 members of the TLR family and selected these five members based on their potential role in autoantibody production. Brentano and colleagues showed recently that *TLR3 *expression was high in RA synovium and increased further after stimulation by TLR3 ligand poly(I–C) or by necrotic RA synovial fluid cells [41]. These data suggest that studies of *TLR3 *polymorphism might be of interest.

## Conclusion

Our study rules out a major contribution of the tested *TLR *polymorphisms to RA in French Caucasians. Our findings need to be confirmed in other cohorts, but already add to the publicity of available data. As we did not observe a large effect it seems that an association between polymorphisms of other *TLR *genes and RA and/or a functional role for *TLR *genes in the pathogenesis of RA would be weakly predictable.

## Abbreviations

anti-CCP: anti-cyclic citrullinated peptide; IL: interleukin; PCR: polymerase chain reaction; RA: rheumatoid arthritis; RF: rheumatoid factor; RFLP: restriction fragment length polymorphism; SNP: single nucleotide polymorphism; TDT: transmission disequilibrium test; TLR: Toll like-receptor.

## Competing interests

The authors declare that they have no competing interests.

## Authors' contributions

OJ and HK performed study design, data analysis, manuscript drafting, and data acquisition. PA, EPT, and GF performed study design, data analysis, and manuscript drafting. CP performed data acquisition. LS performed data analysis and manuscript drafting. MCB and FC performed manuscript drafting.
